# Gastric and Pancreatic Ectopic Mucosa in the Gallbladder: A Unique Mimicker of Polypoid Lesions

**DOI:** 10.7759/cureus.69231

**Published:** 2024-09-11

**Authors:** Ahmet Erbagci, Pınar Engin Zerk

**Affiliations:** 1 Pathology, Istanbul Medeniyet University, Goztepe Prof. Dr. Suleyman Yalcin City Hospital, Istanbul, TUR

**Keywords:** gastroenterology, polyp, heterotopic gastric mucosa, heterotopic pancreas, gallbladder

## Abstract

A 46-year-old woman with upper quadrant pain and nausea, diagnosed with cholelithiasis, underwent cholecystectomy. A 0.7 cm polypoid lesion in the gallbladder showed mostly heterotopic gastric mucosa with antral and oxyntic glands and foveolar epithelium. A focal area of the heterotopic pancreas, comprising acini and ducts with positive trypsin staining but no islet cells, was found. Additional findings included minimal inflammation, an adenomyoma on the opposite wall, and black gallstones, leading to a diagnosis of a heterotopic polyp. This case underscores the importance of thorough histopathological examination in diagnosing rare heterotopic tissues in the gallbladder, preventing misdiagnosis with malignant entities.

## Introduction

Heterotopia is defined as the presence of an organ's tissue in an unusual location within the body, where it maintains its normal histological structure but is not found in its typical anatomical site [1]. This condition often arises congenitally and is most often discovered incidentally. Gastric and pancreatic heterotopia stand as the most prevalent types of heterotopic tissue within the gastrointestinal (GI) tract [2,3]. Gastric heterotopia can occur throughout the entire GI tract, ranging from the tongue down to the rectum [4,5]. It is most commonly localized in the upper portions of the intestine [6]. However, its presence in the gallbladder is exceedingly rare, whereas instances of other varieties of heterotopic tissues in the gallbladder, including liver tissue as well as adrenal and thyroid tissues, have been documented [7]. Also, pancreatic heterotopia can be observed throughout the entire GI tract but is commonly located in the stomach, duodenum, and upper jejunum [8]. However, it is rarely encountered in the gallbladder. Here, we are presenting an incidentally detected case that includes both gastric and pancreatic heterotopias in the gallbladder, a condition reported very rarely in the literature [9].

## Case presentation

**Figure 1:**
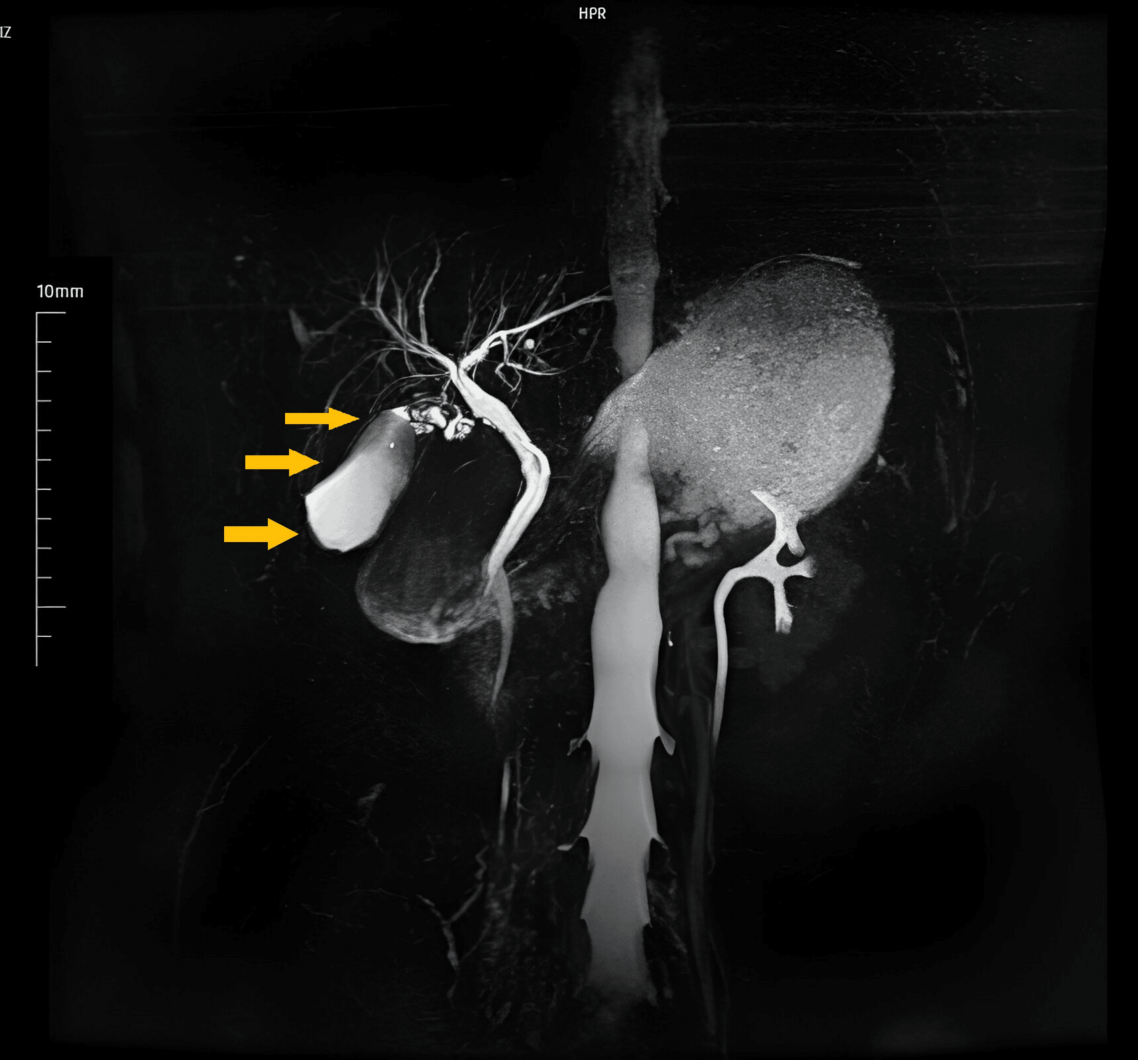
MR cholangiography Multiple millimetric stones in the gallbladder, accompanied by wall thickening and distension revealed.

A 46-year-old female patient presented to our clinic with complaints of right upper quadrant abdominal pain and nausea. Ultrasonography and magnetic resonance cholangiography revealed distension and thickening of the gallbladder wall, along with several millimetric stones (Figure 1), leading to a diagnosis of acute cholecystitis. Laparoscopic cholecystectomy was performed, and gross examination showed a gallbladder measuring 7x3 cm with a smooth serosal surface. The thickness of the wall measured 0.8 cm at its thickest point. Upon opening, the gallbladder revealed five black-colored stones, the largest measuring 5 mm, alongside a green, velvety mucosa with focal hemorrhagic areas. A distinct 0.7 cm polypoid lesion with a smooth contour was also identified and fully sampled for further examination.

**Figure 2:**
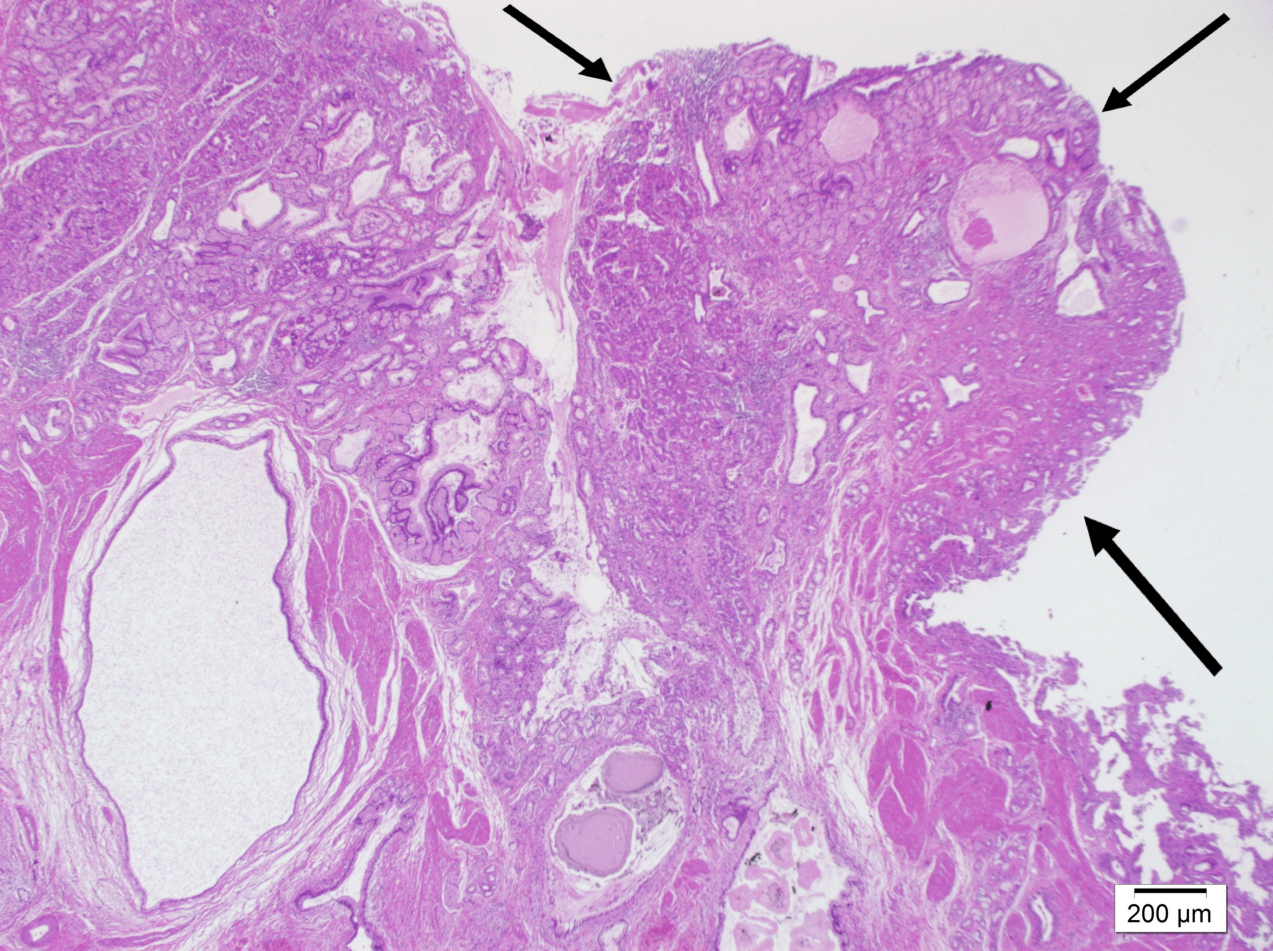
Polypoid lesion at 40x magnification using hematoxylin and eosin staining.

**Figure 3:**
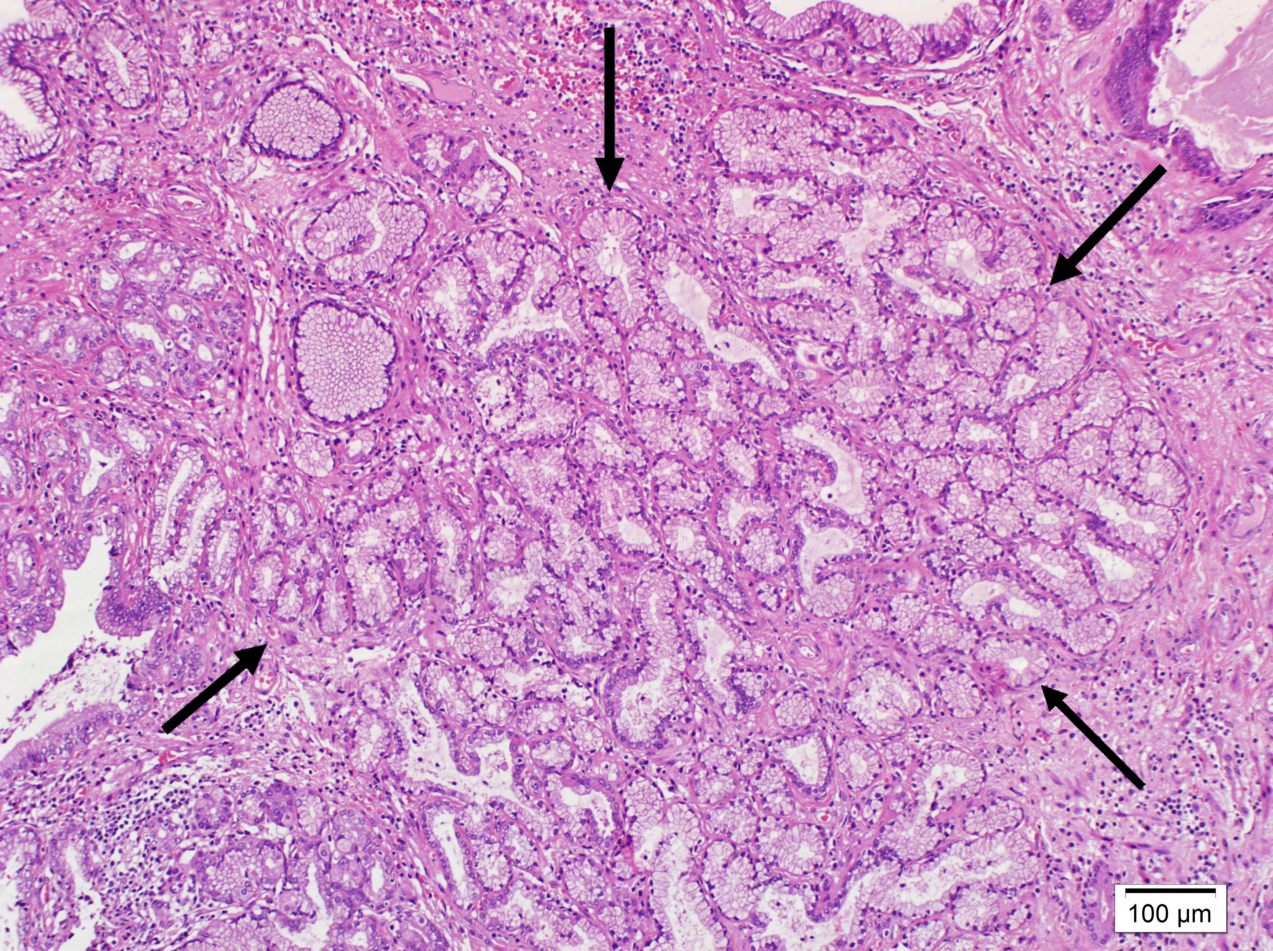
Pyloric glands at 100x magnification using hematoxylin and eosin staining.

**Figure 4:**
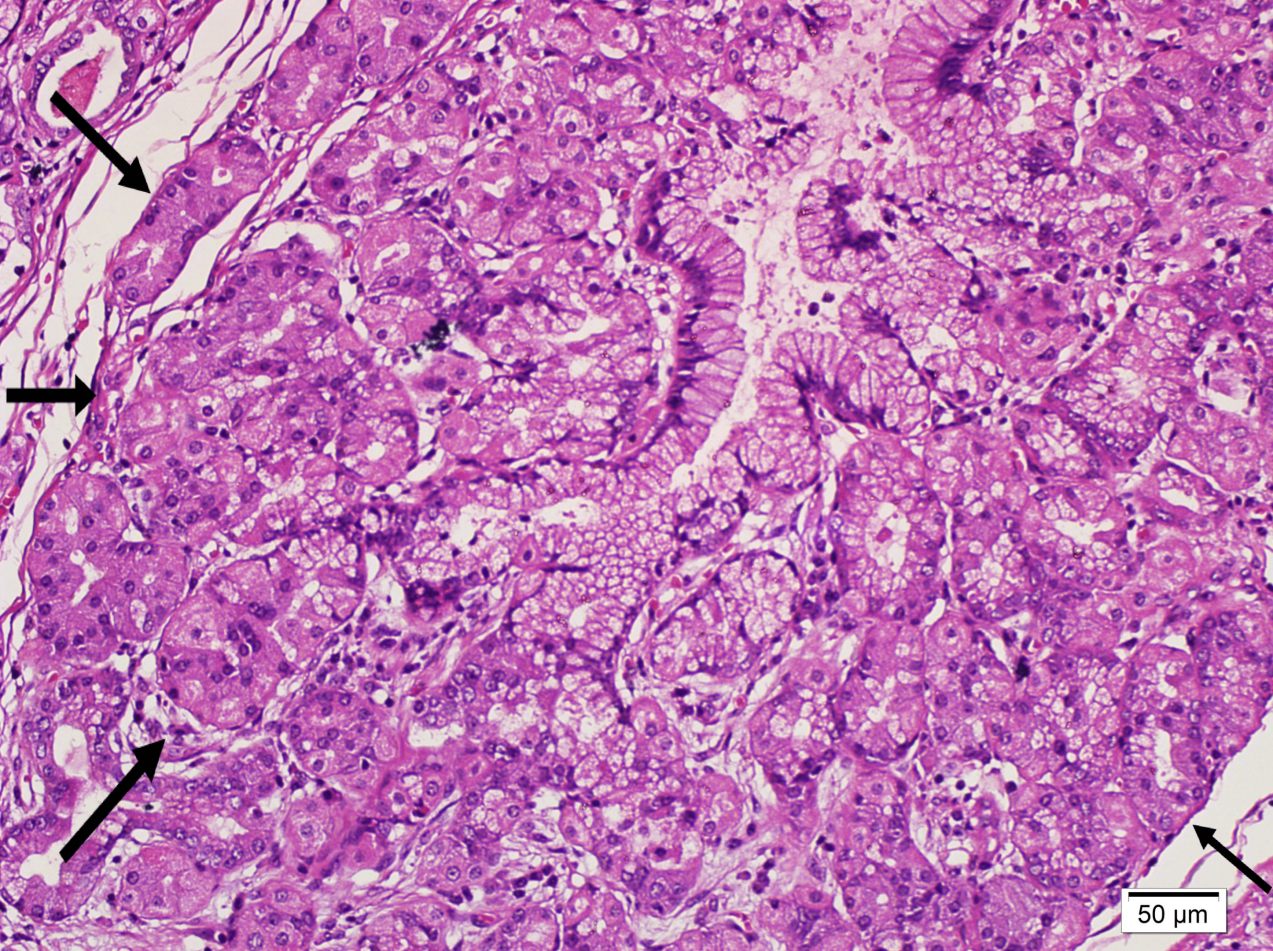
Oxyntic glands at 200x magnification using hematoxylin and eosin staining.

**Figure 5:**
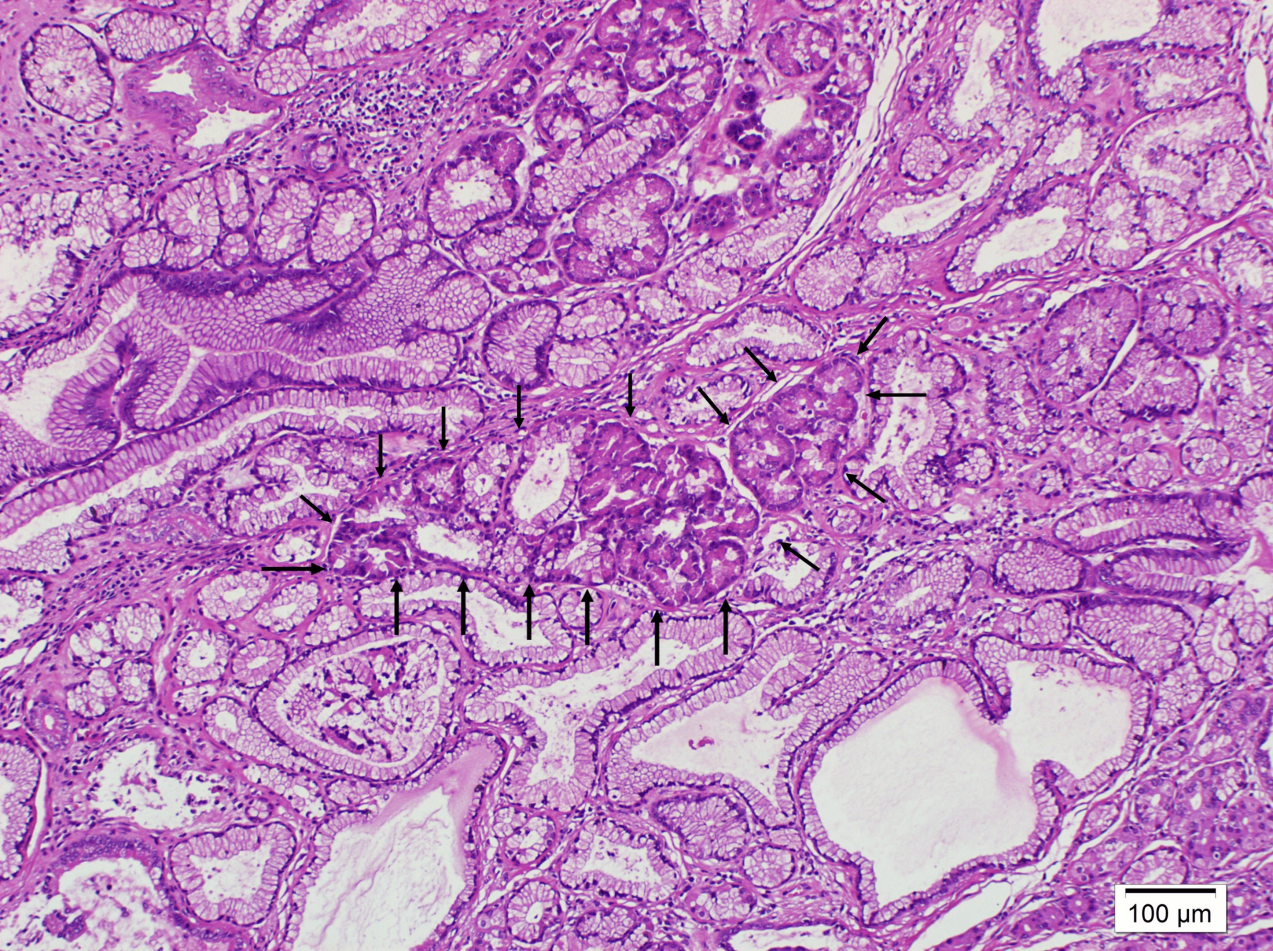
Pancreatic acini and ducts at 100x magnification using hematoxylin and eosin staining.

**Figure 6:**
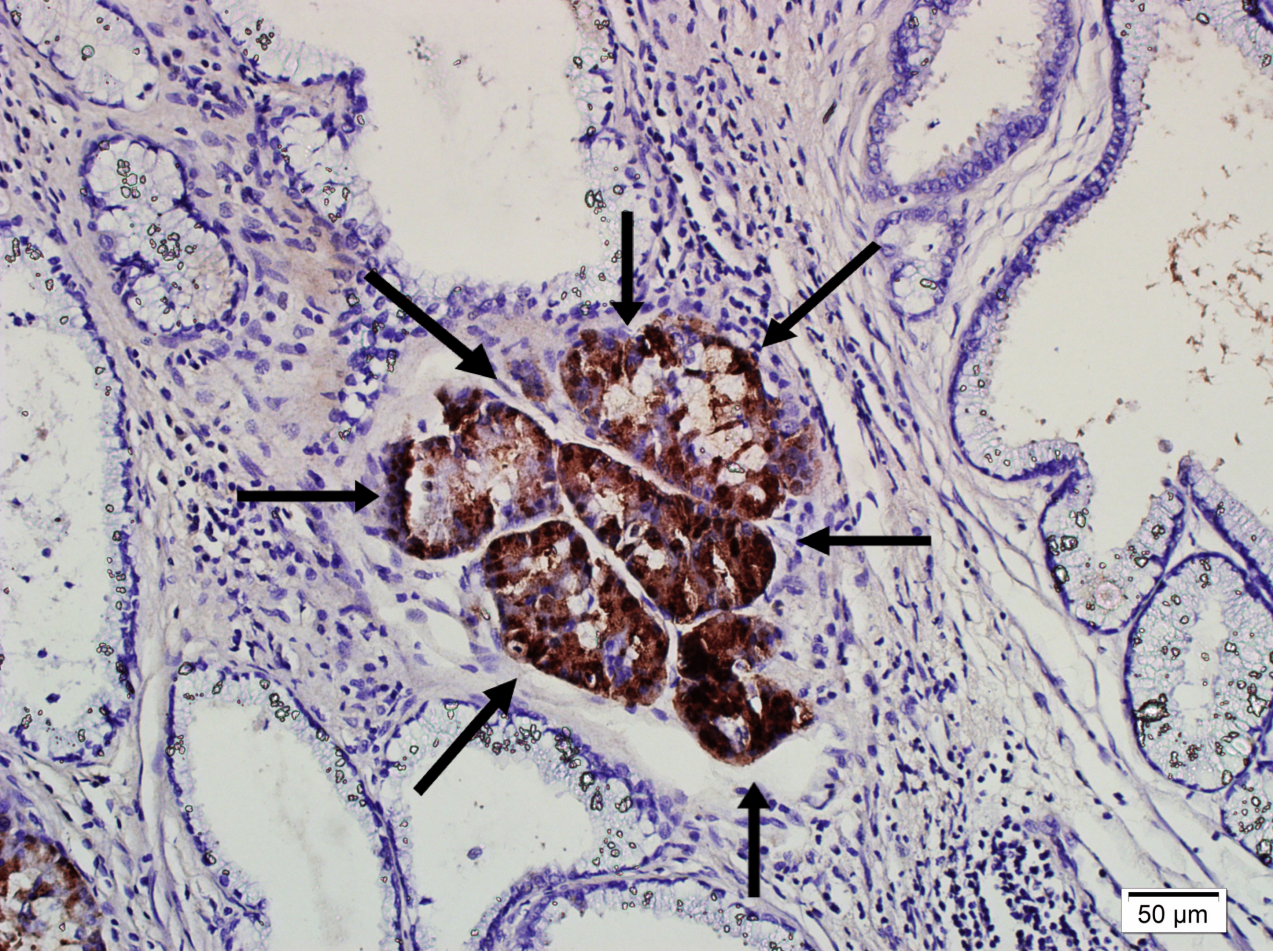
Pancreatic acini positive with trypsin stain at 200x magnification.

Microscopic examination revealed chronic cholecystitis and areas of pyloric metaplasia in the mucosa, alongside a region of adenomyoma. The polypoid lesion (Figure 2) was composed of pyloric-type glands containing neutral mucin (Figure 3), surrounded by oxyntic mucosa consisting of parietal and chief cells and also foveolary-type epithelium (Figure 4). In close proximity to these described areas, pancreatic acini with ducts lacking islets (Figure 5) were observed. These acini showed strong staining with trypsin (Figure 6).

During the perioperative period, ampicillin/sulbactam was administered, and no complications were observed. On follow-up, the patient reported no recurrence of symptoms, and imaging studies showed no evidence of residual or recurrent disease.

## Discussion

Heterotopic gastric and pancreatic tissues are rare anomalies, especially in the gallbladder, where their presence can pose significant diagnostic challenges due to their asymptomatic nature and incidental discovery during routine surgeries. While heterotopic gastric tissue is more frequently encountered in various parts of the GI tract, its coexistence with pancreatic heterotopia in the gallbladder is exceedingly rare. Our case contributes to the limited literature on this rare phenomenon by presenting a unique instance of both gastric and pancreatic heterotopias in the gallbladder.

Previous studies, such as those by Pendharkar et al., have documented cases of either gastric or pancreatic heterotopia in the gallbladder but seldom both. These cases underscore the importance of considering heterotopia in the differential diagnosis when encountering unusual polypoid lesions in the gallbladder, as these lesions can mimic more serious conditions such as gallbladder carcinoma ​[1].

The embryological origin of these heterotopias is thought to result from the misplacement of tissue during the developmental stages of the GI tract. This theory is supported by the close developmental relationship between the stomach and the biliary tree, both of which arise from the endoderm of the primitive intestine ​[7]. Our findings align with this theory, highlighting the necessity of a thorough histopathological examination to differentiate benign heterotopic tissues from potentially malignant lesions.

In addition, imaging techniques, while helpful, often fail to distinguish between benign heterotopias and malignant conditions preoperatively. This limitation underscores the critical role of histological examination in providing a definitive diagnosis ​[7]. Our case further emphasizes the need for pathologists to be aware of the potential for such heterotopias in the gallbladder, particularly in patients presenting with cholelithiasis and other gallbladder-related symptoms.

## Conclusions

This case highlights the rarity of concurrent gastric and pancreatic heterotopias in the gallbladder, which can closely mimic polypoid lesions, potentially leading to diagnostic confusion. Thorough histopathological examination, including adequate sampling, is essential to differentiate these benign lesions from more serious conditions, such as intracholecystic papillary neoplasms and gallbladder carcinoma. Recognizing these heterotopic tissues ensures appropriate treatment, with cholecystectomy being curative and preventing unnecessary aggressive interventions.
